# Association of Uric Acid with Metabolic Syndrome in Men, Premenopausal Women and Postmenopausal Women

**DOI:** 10.3390/ijerph110302899

**Published:** 2014-03-10

**Authors:** Yongqiang Li, Shanying Chen, Xiaofei Shao, Jia Guo, Xinyu Liu, Aiqun Liu, Ying Zhang, Honglei Wang, Bin Li, Kangping Deng, Qin Liu, Harry Holthöfer, Hequn Zou

**Affiliations:** 1 Department of Nephrology, Institute of Nephrology and Urology, The Third Affiliated Hospital of Southern Medical University, Guangzhou 510630, China; E-Mails: liyongqiang851@163.com (Y.L.); shaoxfei@126.com (X.S.); guojia199683@126.com (J.G.); pepsi84@163.com (X.L.); nanyitx@126.com (A.L.); xiaoying2001-205@163.com (Y.Z.); ailemonmint@126.com (H.W.); libin12014@163.com (B.L.); kangpingdeng82@163.com (K.D.); aiq2006@126.com (Q.L.); 2 Department of Nephrology, Zhangzhou Affiliated Hospital of Fujian Medical University, Zhangzhou 363000, China; E-Mail: dqydqy386@sina.com; 3 National Centre for Sensor Research/BioAnalytical Sciences, Dublin City University, Dublin 9, Ireland; E-Mail: harry.holthofer@dcu.ie

**Keywords:** uric acid, metabolic syndrome, premenopausal women and postmenopausal women

## Abstract

*Objective*: To explore the relationship between serum uric acid (SUA) and metabolic syndrome (MS) in men, premenopausal women and postmenopausal women. *Methods*: A cross-sectional study was conducted in 1,834 community-based Southern Chinese participants from June to October 2012. Sex-specific SUA quartiles were used as follows: <345, 345–<400, 400–<468, ≥468 µmol/L in males; and <248, 248–<288, 288–<328, ≥328 µmol/L in females. MS was defined by the National Cholesterol Education Program Adult Treatment Panel III (NCEP ATP III) Criteria. The association between SUA and MS was then analyzed using the STATA software. *Results*: The odds ratio (OR) for having MS in the highest *versus* lowest quartiles of SUA levels was 2.46 (95% confidence interval [CI], 1.39 to 4.34, *p* = 0.002) in men after adjusting for age, sex, history of coronary heart disease, history of stroke, current current smoking, current alcohol use, physical inactivity, education status, and BMI. Further adjusting for above confounders, hypertension and diabetes, the OR for having MS in the highest *versus* lowest quartiles of SUA was 3.06 (95% CI, 1.64 to 5.70, *p* < 0.001). The OR for having MS in the highest *versus* lowest quartiles of SUA was 3.45 (95% CI, 1.38 to 8.64, *p* = 0.008) and 1.98 (95% CI, 1.16 to 3.37, *p* = 0.08) in premenopausal women and postmenopausal women after adjusting for age, sex, history of coronary heart disease, history of stroke, current smoking, current alcohol use, physical inactivity, education status, and BMI. Further adjusting for above confounders, hypertension and diabetes, the OR for having MS in the highest *versus* lowest quartiles of SUA was 3.42 (95% CI, 1.15 to 10.18, *p* = 0.03) and 1.87 (95% CI, 1.05 to 3.33, *p* = 0.03) in premenopausal women and postmenopausal women. *Conclusions*: Higher SUA levels are positively associated with the presence of MS in males and females. Higher SUA levels had a higher risk of having MS in premenopausal women than in postmenopausal women.

## 1. Introduction

Uric acid (UA) is a metabolic product of purine. Hyperuricemia has been associated with several metabolic and cardiovascular conditions, including diabetes and coronary artery disease [1,2,3]. Metabolic syndrome (MS) designates a group of cardiac risk factors consisting of insulin resistance (IR) (impaired insulin action), visceral obesity, atherogenic dyslipidemia, endothelial dysfunction, and systemic inflammation [4]. It has been well established that MS exposes one to numerous cardiovascular risks [5,6,7].

Some large epidemiologic studies have shown that the prevalence of MS was positively related to serum levels of UA (SUA) [8,9,10]. Menopause is independently associated with SUA levels, whereas postmenopausal hormone use is associated with lower UA levels among postmenopausal women [11]. Postmenopausal status is associated with an increased risk of MS [12]. To the best of our knowledge, few studies have assessed the relationship between MS and UA regarding menopausal status and gender. Thus, this purpose of this study was to determine the association between MS and UA according to menopausal status. In addition, we explored this association in men of this population.

## 2. Methods

### 2.1. Participants

Data were drawn from a population-based, cross-sectional survey conducted in Wanzhai Town, Zhuhai City, a prominent commercial city in Southern China. Zhuhai is located on the southern coast of China. Participants were selected using a multi-stage stratified random cluster sampling method. Step 1, two communities were selected randomly from Wanzhai Town; Step 2, in each of the 2 selected communities, 500 families were randomly sampled as the target family; and Step 3, all the residents aged from 18 to 75 in the selected families were sampled. Using this method, a total of 2,142 participants from 2,603 residents completed the survey, with a response rate of 82.2%. Participants were recruited by mail and home visits. First, we informed participants by mail. Then we visited the families and retrieved the questionnaires. This survey was conducted between June 2012, and October 2012. The Ethics Committee of The Third Affiliated Hospital of Southern Medical University, Guangzhou, approved this study. All community residents gave their written informed consent. Details of this cross-sectional study has been described in our previous paper [13].

### 2.2. Study Measurements

Socio-demographic characteristics, including personal health history (coronary artery disease, stroke, hypertension, and diabetes) and details about lifestyle (smoking status, alcohol intake, diet habits and physical activity) were obtained by questionnaire. Education status was classified into two categories: (1) 0 years of schooling, primary school or junior middle school; (2) high school or above [13,14]. Body weight, height, waist circumference and blood pressure were measured with standardized protocol in the morning between 08:00 a.m. and 11:00 a.m. Central obesity was defined by waist measurement >90 cm for men or >80 cm for women [15]. Blood pressure was measured twice to the nearest 2 mmHg by a trained nurse using a mercury totally closed desk-top sphygmomanometer (Model XJ300/40-1, Shanghai, China), after the participants had been seated at least for 5 min. The first and fourth Korotkoff sounds were used to represent the systolic blood pressure (SBP) and diastolic blood pressure (DBP). The average value of these two measuring points for systolic and diastolic blood pressure was recorded. Blood glucose level was measured with a hexokinase enzyme reference method and serum creatinine (SCr) with an enzymatic method on an autoanalyzer (Hitachi 7170, Hitachi, Tokyo, Japan). SUA level was measured with a colorimetric method (Roche Diagnostics, Mannheim, Germany). Serum high-density lipoprotein (HDL) was determined enzymatically with commercially available reagents (Shanghai Gensource Co., Ltd, Shanghai, China), and cholesterol and triglyceride (TG) levels were determined enzymatically with commercially available reagents (Roche Diagnostics). Low density lipoprotein cholesterol (LDL-C) was measured by the Friedewald formula. High sensitivity C-reactive protein was measured by enzymatic Turbidimetric immunoassay method (Orion Diagnostica Oy, Espoo, Finland). The estimated glomerular filtration rate (eGFR), an indicator of kidney function, was estimated using a formula from the Chinese-Modification of Diet Renal Disease (C-MDRD) study: GFR (mL/min/1.73 m^2^) = 175 × (Scr) − 1.234 × (Age) − 0.179 × (if female, ×0.79) [16].

### 2.3. Determination of Hyperuricemia

Hyperuricemia was defined as SUA ≥ 7 mg/dL in men or ≥6 mg/dL in women [17,18]. SUA levels were divided into separate quartiles for males and females. Sex-specific SUA quartiles were used as follows: <345, 345–<400, 400–<468, ≥468 µmol/L in males; <248, 248–<288, 288–<328, ≥328 µmol/L in premenopausal women; and <281, 281–<329, 330–<380, ≥328 µmol/L in postmenopausal women. 

### 2.4. Determination of MS

MS was determined according to the criteria of the NCEP ATP III. Thus, MS was defined as the presence of three or more of the following five criteria: (1) waist circumference ≥ 90 cm in males and ≥80 cm in females; (2) triglycerides ≥ 150 mg/dL or under treatment for elevated triglycerides; (3) high-density lipoprotein (HDL)-cholesterol < 40 mg/dL in males and <50 mg/dL in females or under treatment for reduced HDL; (4) SBP ≥ 130 mmHg or DBP ≥ 85 mmHg or under treatment for hypertension; and (5) fasting glucose ≥ 100 mg/dL or under treatment for elevated glucose [19].

### 2.5. Determination of Hypertension and Diabetes

Systolic BP ≥ 140 mmHg or diastolic BP ≥ 90 mmHg or under treatment for hypertension diagnosed for hypertension. Fasting glucose ≥ 7.0 mmol/L or under treatment for treatment for previously diagnosed diabetes diagnosed for diabetes.

### 2.6. Menopausal Status

According to an epidemiological survey in Guangdong Province (Zhuhai is one of cities in Guangdong Province), the averagenope of natural mause is 48.9 years in women from the urban areas [20]. Women older than 48.9 years were classified as postmenopausal status and others were classified as premenopausal status. 

## 3. Statistical Analysis

Data were analyzed using STATA (version 11, Stata Press, College Station, TX, USA). Mean ± standard deviation (SD) was reported for numerical variable. Proportions were reported for categorical variables.

All participants were divided into three subgroups: men, premenopausal and postmenopausal women. SUA levels were divided into quartiles. Baseline characteristics of four quartiles subjects were examined in the three subgroups. The continuous variables were analyzed by Wilcoxon rank-sum test and the categorical variables were analyzed by the chi-squared test or Fisher’s exact test. 

Logistic regression models were used to examine whether UA is associated MS in men, premenopausal and postmenopausal women. SUA was divided into quartiles and used as an independent variable. Model one was adjusted for lifestyle factors (current smoking, alcohol use, physical inactivity), age, sex, comorbidities (history of coronary heart disease, history of stroke), education status and BMI were included. To examine whether hypertension and diabetes are in the pathway between SUA and MS, hypertension and diabetes were included in the next model. The lowest quartile group was a reference category. Logistic regression analyses were conducted separately in men, premenopausal and postmenopausal women. All statistical tests were 2-sided, and *p* < 0.05 was considered statistically significant.

## 4. Results

There were 2,142 study subjects (the mean age was 49.55 ± 13.44 years, 796 were men and all participants were of the Han ethnicity). Three hundred and eight (308) participants were excluded because of missing data for SUA, serum creatinine or anthropometric. We included 1,834 participants with mean age 52.7 ± 14.6 years in our study. Among them, 679 were men (37.02%) and 1,155 (62.98%) were women. Six hundred and fifty seven (657) women older than 48.9 years were classified as postmenopausal status and 498 women were classified as premenopausal status.

### 4.1. Baseline Characteristics of Three Subgroups

As shown in Table 1, Table 2 and Table 3, in men, premenopausal and postmenopausal women subgroups, subjects with the higher quartile SUA had a higher BMI, a larger waist circumference, higher systolic blood pressure, higher diastolic blood pressure. And, subjects with the higher quartile SUA also had higher serum creatinine levels, higher CRP levels, higher triglyceride levels and lower eGFR. These differences were significant (*p* < 0.05). 

**Table 1:** Baseline characteristics of male subjects.

Characteristic	Quartile one	Quartile two	Quartile three	Quartile four	*p* value
	<345	>345 & <400	>400 & <468	>468	
	*N* = 170	*N* = 170	*N* = 170	*N* = 169	
Serum uric acid (umol/L)	306 (217–329)	373 (361–383)	432 (414–448)	519 (489–561)	<0.001
Clinical Characteristics					
Age (Years)	54.12 ± 12.93	51.72 ± 15.04	53.59 ± 15.6	53.51 ± 16.06	0.03
Hypertension	71	66	75	87	0.10
Diabetes	21	11	11	19	0.12
Current smoker (%)	69	43	49	53	0.01
Current alcohol use (%)	23	19	25	27	0.62
High school or above (%)	78	99	82	85	0.09
Physical inactivity (%)	106	86	89	93	0.16
Systolic blood pressure (mm Hg)	128.65 ± 18.76	126.36 ± 17.85	132.78 ± 19.85	133.99 ± 19.68	0.001
Diastolic blood pressure (mm Hg)	78.78 ± 9.57	77.95 ± 11.30	80.15 ± 10.74	81.65 ± 10.57	0.03
Body Mass Index (kg/m^2^)	23.06 ± 3.24	23.58 ± 3.58	23.79 ± 3.21	24.80 ± 3.26	<0.001
Waist circumference (cm)	84.14 ± 9.46	85.69 ± 9.72	86.52 ± 9.46	89.60 ± 9.16	<0.001
Laboratory values					
Serum Creatitine (umol/L)	82.77 ± 13.27	84.32 ± 13.05	88.05 ± 15.34	93.68 ± 16.91	<0.001
eGFR(mL/min/1.73m^2^)	96.87 ± 19.90	95.31 ± 18.02	90.37 ± 18.44	84.80 ± 21.49	<0.001
Fasting glucose (mmo/L)	5.31 ± 1.73	4.94 ± 1.12	4.93 ± 0.97	5.11 ± 1.05	0.046
Serum triglyceride (mmol/L)	1.22 (0.88–1.76)	1.15 (0.88–1.82)	1.40 (1.04–2.14)	1.9 (1.25–2.62)	<0.001
Serum low density lipoprotein (mmol/L)	3.14 ± 0.88	3.15 ± 0.82	3.14 ± 0.94	3.13 ± 1.03	0.98
Serum high density lipoprotein (mmol/L)	1.48 ± 0.33	1.44 ± 0.29	1.41 ± 0.33	1.41 ± 0.31	0.19
Serum C-reactive protein (mg/L)	1.01 (0.49–2.63)	0.67 (0.32–1.65)	1.25 (0.62–2.76)	<0.001	<0.001

Mean ± SD or median (25th to 75th percentiles) for continuous variables and proportion (95% confidence interval) for category variables are presented.

**Table 2:** Baseline characteristics of premenopausal women.

Characteristic	Quartile one	Quartile two	Quartile three	Quartile four	*p* value
	<248	248–288	288–328	>328	
	*N* = 125	*N* = 124	*N* = 125	*N* = 124	
Serum uric acid (umol/L)	249 (229–265)	303 (293–316)	351 (339–363)	445 (403–487)	<0.001
Clinical Characteristics					
Age (Years)	60.92 ± 7.88	61.59 ± 8.30	62.55 ± 8.32	65.12 ± 8.65	<0.001
Hypertension	78	72	99	108	<0.001
Diabetes	14	18	22	25	0.22
Current smoker (%)	1	1	1	0	0.86
Current alcohol use (%)	3	1	1	3	0.60
High school or above (%)	42	42	26	39	0.14
Physical inactivity (%)	84	76	76	59	0.06
Systolic blood pressure (mm Hg)	131.55 ± 19.93	131.45 ± 19.25	138.76 ± 23.01	140.16 ± 17.68	0.01
Diastolic blood pressure (mm Hg)	77.92 ± 10.02	76.68 ± 10.19	81.30 ± 11.99	80.22 ± 10.37	0.003
Body Mass Index (kg/m^2^)	21.43 ± 2.46	22.42 ± 2.89	22.23 ± 3.33	22.88 ± 3.86	0.02
Waist circumference (cm)	80.71 ± 8.61	81.85 ± 9.23	85.53 ± 9.13	87.24 ± 10.07	0.07
Laboratory values					
Serum Creatitine (umol/L)	61.95 ± 8.31	65.56 ± 9.83	67.80 ± 9.73	73.19 ± 14.00	<0.001
eGFR (mL/min/1.73m^2^)	105.56 ± 18.06	98.68 ± 17.83	94.33 ± 17.43	86.63 ± 18.87	<0.001
Fasting glucose (mmo/L)	4.99 ± 1.17	5.11 ± 1.22	5.57 ± 1.57	5.35 ± 1.30	0.36
Serum triglyceride (mmol/L)	1.12 (0.84–1.43)	1.24 (0.922–1.73)	1.42 (1.06–1.96)	1.51 (1.14–2.28)	<0.001
Serum low density lipoprotein (mmol/L)	3.23 ± 0.87	3.48 ± 0.88	3.51 ± 0.89	3.62 ± 0.92	0.07
Serum high density lipoprotein (mmol/L)	1.67 ± 0.35	1.65 ± 0.37	1.55 ± 0.30	1.53 ± 0.28	0.06
Serum C-reactive protein (mg/L)	0.84 (0.45–1.82)	1.05 (0.50–2.05)	1.45 (0.71–3.23)	2.07 (0.89–4.80)	0.001

Mean ± SD or median (25th to 75th percentiles) for continuous variables and proportion (95% confidence interval) for category variables are presented.

**Table 3:** Baseline characteristics of postmenopausal women.

Characteristic	Quartile one	Quartile two	Quartile three	Quartile four	*p* value
	<281	281–329	330–380	>380	
	*N* = 164	*N* = 164	*N* = 164	*N* = 165	
Serum uric acid (umol/L)	225 (205–236)	265 (256–275)	306 (295–317)	368 (345–418)	<0.001
Clinical Characteristics					
Age (Years)	40.54 ± 7.53	39.76 ± 7.97	38.05 ± 8.80	38.76 ± 9.14	<0.001
Hypertension	25	20	26	38	0.04
Diabetes	2	4	0	4	0.18
Current smoker (%)	1	3	1	0	0.35
Current alcohol use (%)	0	2	0	2	0.27
High school or above (%)	68	65	74	61	0.39
Physical inactivity (%)	84	90	83	87	0.53
Systolic blood pressure (mm Hg)	114.39 ± 13.61	114.27 ± 15.23	117.23 ± 15.42	121.14 ± 18.03	<0.001
Diastolic blood pressure (mm Hg)	72.18 ± 9.65	72.56 ± 9.17	74.66 ± 9.87	78.21 ± 12.59	<0.001
Body Mass Index (kg/m^2^)	21.43 ± 2.46	22.42 ± 2.89	22.23 ± 3.33	22.88 ± 3.86	<0.001
Waist circumference (cm)	75.46 ± 7.05	77.19 ± 7.42	77.46 ± 8.97	78.72 ± 9.94	<0.001
Laboratory values					
Serum Creatitine (umol/L)	58.207 ± 7.09	61.27 ± 8.70	63.45 ± 7.91	65.16 ± 9.23	<0.001
eGFR (mL/min/1.73m^2^)	122.45 ± 19.97	116.43 ± 22.94	111.69 ± 18.37	108.40 ± 20.20	<0.001
Fasting glucose (mmo/L)	4.66 ± 0.87	4.71 ± 0.87	4.60 ± 0.41	4.75 ± 0.76	<0.001
Serum triglyceride (mmol/L)	0.82 (0.63–1.09)	0.97 (0.72–1.32)	0.96 (0.69–1.38)	1.13(0.84–1.82)	<0.001
Serum low density lipoprotein (mmol/L)	2.86 ± 0.74	2.71 ± 0.76	2.90 ± 0.77	2.97 ± 0.81	<0.001
Serum high density lipoprotein (mmol/L)	1.64 ± 0.28	1.59 ± 0.32	1.58 ± 0.31	1.56 ± 0.35	<0.001
Serum C-reactive protein (mg/L)	0.45 (0.25–1.03)	0.77 (0.37–1.77)	0.73 (0.32–1.70)	0.85 (0.39–1.75)	<0.001

Mean ± SD or median (25th to 75th percentiles) for continuous variables and proportion (95% confidence interval) for category variables are presented.

### 4.2. Associations of SUA with MS in Men, Premenopausal and Postmenopausal Women

As shown in Table 4, After adjustment for age, comorbidities (history of coronary heart disease, history of stroke), lifestyle factors (current smoking, alcohol use, physical inactivity), education status and BMI, SUA was significantly associated with MS in men (OR 2.46, 95% CI 1.39, 4.34, *p* = 0.002, comparing the highest to the lowest quartile). After controlling for hypertension and diabetes, the association of SUA with MS was still significant. Men with the highest quartile of SUA had an increased risk for MS (OR 3.06, 95% CI 1.64, 5.70, *p* < 0.001), Compared with the lowest quartile of SUA. Men with the third and the second quartiles of SUA also had an increased risk for MS, but the difference was not significant. 

**Table 4:** Association of uric acid andmetabolic syndrome in men.

Quintiles of SUA	Model one ^a^		Model two ^b^	
	OR (95% CI)	*p* value	OR (95% CI)	*p* value
Quartile one	Reference		Reference	
Quartile two	0.81	0.51	1.10	0.78
	(0.43, 1.53)		(0.56, 2.18)	
Quartile three	1.00	1.00	1.37	0.35
	(0.55, 1.83)		(0.71, 2.65)	
Quartile four	2.46	0.002	3.06	<0.0001
	(1.39, 4.34)		(1.64, 5.70)	

^a^ Adjusted for age, sex, history of coronary heart disease, history of stroke, current smoker, current alcohol use, physical inactivity, education attainment, and BMI; ^b^ Adjusted for above + hypertension, diabetes.

As shown in Table 5, in both premenopausal and postmenopausal women, women with higher quartiles of SUA had an increased risk for MS comparing to women with the lowest quartile SUA. But the difference was only significant in the highest quartile of uric acid. An increased risk for MetS in the highest quartile SUA was higher in premenopausal women than postmenopausal women. Comparing the highest to the lowest quartile, OR were 3.42 (95% CI, 1.15 to 10.18, *p* = 0.03) and 1.87 (95% CI, 1.05 to 3.33, *p* = 0.03), respectively.

**Table 5:** Association of uric acid and metabolic syndrome in premenopausal and postmenopausal women.

Quintiles of SUA	Premenopausal women				Postmenopausal women			
	Model one ^a^		Model two ^b^		Model one ^a^		Model two ^b^	
	OR (95% CI)	*p* value	OR (95% CI)	*p* value	OR (95% CI)	*p* value	OR (95% CI)	*p* value
Quartile one	Reference		Reference		Reference		Reference	
Quartile two	1.13	0.81	1.22	0.33	1.34 (0.79, 2.28)	0.28	1.39	0.27
	(0.42, 3.08)		(0.36, 4.12)				(0.78, 2.46)	
Quartile three	1.61	0.34	2.24	0.71	1.50 (0.88, 2.54)	0.13	1.30	0.36
	(0.61, 0.25)		(0.73, 6.84)				(0.74, 2.30)	
Quartile four	3.45	0.008	3.42	0.03	1.98 (1.16, 3.37)	0.01	1.87	0.03
	(1.38, 8.64)		(1.15, 10.18)				(1.05, 3.33)	

^a^ Adjusted for age, sex, history of coronary heart disease, history of stroke, current smoker, current alcohol use, physical inactivity, education attainment, and BMI; ^b^ Adjusted for above + hypertension, diabetes.

## 5. Discussion and Conclusions

To the best of our knowledge, the current study is the first to evaluate the relationships of SUA levels with MS in a Chinese population according to menopausal status and gender. We found that there is a significant association between SUA and MS in men, premenopausal women and postmenopausal women. And this association was independent of age, sex, comorbidities (history of coronary heart disease, history of stroke), lifestyle factors (current smoking status, current alcohol use, physical inactivity), education status, BMI, hypertension and diabetes. Furthermore, we found the risk of MS to be higher in premenopausal women than in postmenopausal women in the highest quartile of the SUA group. Comparing the highest to the lowest quartile, OR were 3.42 (95% CI 1.15–10.18, *p* = 0.03) and 1.87 (95% CI 1.05–3.33, *p* = 0.03), respectively.

As a proved risk factor of hypertension, dyslipidemia and hyperuricemia, obesity plays an important role in the increasing prevalence of MS [21]. In our study population, SUA levels were significantly and positively associated with waist circumference and BMI in men as well as in premenopausal women and postmenopausal women. These relationships are consistent with those of other studies [8,22,23].

Previous studies reported that SUA is more closely associated with impaired fasting glucose and diabetes in women than in men [24,25]. No association between SUA and fasting glucose was found in men in our study. This result is consistent with previous studies [24,25]. We further analyzed this association in women according to menopausal status. This led us to find a significant association in postmenopausal women, but not in premenopausal women. However, the reason for this finding remains unclear. 

SUA levels were significantly and positively associated with systolic blood pressure, diastolic blood pressure, serum C-reactive protein, serum triglyceride, and significantly and negatively associated with eGFR in every sub-population in our study. These results are, in general, consistent with previous reports [26,27,28,29].

The association between SUA and MS has obtained much attention in recent years. Previous studies, including cross-sectional [27,30] and prospective studies [31,32], showed that increased serum uric acid levels were associated with increased risk of MS. The findings of our present study are in accordance with them. The underlying mechanisms of the association between SUA levels and MS risk remain poorly understood. Many studies have shown that not only did SUA associate with concomitant insulin action, blood pressure, and lipids, it also predicted future declines in insulin action and T2DM [23,33,34]. Previous studies showed that hyperinsulinemia induces a significant reduction in the urinary excretion of uric acid and sodium [35,36], Insulin resistance and resultant hyperinsulinemia are thought to constitute the pathophysiological cause of MS. In addition, we found a higher risk for MS in premenopausal women than in the group of postmenopausal women in the highest quartile of the SUA group. Comparing the highest and the lowest quartiles, OR were 3.42 (95% CI 1.15–10.18, *p* = 0.03) and 1.87 (95% CI 1.05–3.33, *p* = 0.03) in premenopausal women and postmenopausal women, respectively. The reason for this result remains unclear. Sex hormone function plays a crucial role in menopausal transition for women. Mumford *et al.* [37] reported mean SUA concentrations were highest during the follicular phase, and were inversely associated with endogenous estradiol (E2) and progesterone, and positively associated with FSH. A meta-analysis showed hormone-replacement therapy (HRT) reducing abdominal obesity, insulin resistance, new-onset of diabetes, lipids, blood pressure, adhesion molecules and procoagulant factors in women without diabetes and reduced insulin resistance and fasting glucose in women with diabetes [38]. Thus, further studies are warranted for the female hormonal influence on the association between SUA and MS.

There are several limitations in our study. First, it is difficult to identify the causality between SUA and MS. Second, due to our data limitation, we have to analyze the menopausal status according to the average rate of natural menopause in women from the urban areas of Guangdong Province. Third, we have not measured the levels of female hormones, which might shed more light on the different associations among SUA and MS in the groups of premenopausal women and postmenopausal women.

In summary, our study showed that SUA was associated with presence of MS in men, premenopausal women and postmenopausal women. The risk for MetS was higher in premenopausal women than postmenopausal women in the highest quartile uric acid group. Further studies are warranted for the female hormonal influence on the association between SUA and MS.
